# Bodies, Sport and Science in the Nineteenth Century *

**DOI:** 10.1093/pastj/gtw004

**Published:** 2016-04-19

**Authors:** Vanessa Heggie

**Affiliations:** University of Birmingham

In 1876 the renowned American long-distance pedestrian Edward Payson Weston travelled to Britain to challenge local sportsmen and to raise his transatlantic sporting profile. In February, wearing his distinctive athletic outfit of knickerbockers, leather leggings and walking boots that ‘reach[ed] above the ankle’, he attempted a walk of 115 miles in twenty-four hours around a track in the Royal Agricultural Hall in Islington, London. ^1^ This race is relatively well known in the history of sport, used to demonstrate increasing internationalism, or perhaps increasing professionalization, in sport, both taken as symptoms of modernity. ^2^ My argument is that Weston’s walk should be allowed to intrude on other histories too: he was part of an international debate about science, and a national debate about the relationship between the state and its citizens, which embroiled chemists, physiologists, physicists, doctors and social reformers for years. This iteration of the debate had been sparked in earnest a little over a decade earlier, in 1865, when two German researchers climbed the Faulhorn, one of a ring of mountains in the Bernese Alps, eating only fried starch paste, drinking only sugary tea and meticulously collecting their urine. ^3^

Following the path from the Faulhorn to the Royal Agricultural Hall leads us through the first encounters between modern sports and modern, largely biomedical, sciences. Current historical writing portrays this connection as either non-existent or essentially antagonistic. ^4^ This article takes a contrary position, demonstrating instead how nineteenth-century science and sport came together in mutually beneficial interactions. This process helped to define notions of health, vigour and national identity, as well as solving some crucial scientific puzzles. Here I argue that paying closer attention to actors like Weston shifts our perspective on events like the Royal Agricultural Hall race, and will reveal other important stories: in Weston’s case, the earliest documented doping controversy in modern sport, an event so far entirely overlooked by historians.

Modern sport and modern experimental science are both products of the intellectual and industrial changes that took place in nineteenth-century Europe; they are both nationally specific products, framed by local social and political institutions, while also both participating in an international trade in ideas and ideologies. ^5^ A set of common sociopolitical pressures, outlined below, acted to align the research priorities of science and the practices of sport across Europe. Consequently, both modern science and modern sport could express national strength or reveal national weakness, in physical as well as intellectual or economic terms; both were explicitly framed as processes to reform or preserve national bodies, literally as well as figuratively.

Framed in this perspective, it may seem strange that the relationship between the two histories has not been better studied. In this article two explanations are given for this silence. Firstly, there is the tendency for nineteenth-century writers and thinkers to take the athletic adult male body as a default state, a ‘normal’ that does not merit comment. Sportsmen (and they are all men in this story) are effectively made invisible by this assumption, so that the sporting part of their identity is generally only visible in the relatively rare situations when it is challenged or problematic: when they are being studied in the context of claims that severe sporting exertion is unhealthy, or that their doping has affected a physiologist’s conclusions, or that their bodies are not good models on which to base policy relating to prison diets.

Secondly, then, this variable visibility has led historians to suggest that doctors and scientists in the late nineteenth century were sceptical about the value of sport, or even acted to limit and constrain athletic participation. ^6^ One reason why this view has persisted is that it fits prevailing trends in the historiography of both sport and science. In this article these tendencies are opened out and criticized in more detail, but in brief the issue is that, in the case of sport, amateurism and scientific intervention are considered to be opposed to one another; in the case of science, a prioritization of attention towards laboratory sciences has meant that clinical field studies (and awkward physiologists who do not fit neatly into existing narratives) tend to be ignored. The stories of athletes are most likely to be found in statistical and epidemiological investigations, and in field experiments and clinical case studies, and it is no coincidence that these are exactly the historical practices that tend to be marginalized in the traditional history of biology and medicine, which focuses on the ‘rise of the laboratory’ and the industrialization of science. ^7^

Compounding this situation is the fact that particular stories and publications from the nineteenth century have been cherry-picked and then repeatedly re-cited in historical works, effectively fossilizing into a kind of ‘canon’ on sport and science. ^8^ In this article these works are refigured, and put into a different context, sometimes re-read against the grain of existing histories. This process reveals them as quite different objects, not least because they illustrate a second problem, which is a persistent issue with the way in which technical terminology is read. Phrases such as ‘moderate exercise’ and words like ‘fatigue’ are deceptively complicated, and vary significantly over time; when they are treated bluntly as transhistorical concepts, they distort our understanding of past events. ^9^

The case studies of Weston, and the field study on the Faulhorn, are focal points in this account because they offer a counter-narrative. Here I am going to walk through the story that connects a fried-paste diet to Weston’s doping scandal, and, in doing so, outline the beginning of a new alternative history, one that will properly reconnect sport and science. In the process, I shall also show how these events were laced into bigger stories about bodies, nations, industrial anxiety and welfare reform.

## I

### INVIGORATED SPORT AND DEGENERATE PEOPLES

Before looking at specifics it will be useful to see how the major forms of modern sport were themselves shaped by existing biomedical and socio-political anxieties. Grossly simplified, three new kinds of physical culture dominated northern and western Europe in the nineteenth century: the outdoor athletics of the German *Turnen* , the rhythmic movements and body culture of the Swedish Ling gymnastics, and the team and competitive sports of Victorian Britain. These were combined with, and altered by, one another and by native sports traditions, but despite these variations all three core systems shared two important features. Firstly, all were supposed to have effects on the mind as well as the body, forming characters or instilling appropriate moral virtues (although the precise content of those characters and the nature of the morals were as variable as the systems). Secondly, these systems often reached prominence owing to debates about national strength and national identity, or were fundamentally altered by them. These two features mean that modern sport, exercise and body culture was (and sometimes still is) systematically promoted as a cure for modern ills, particularly national decay and decadence, whether moral, spiritual or physical. When national and racial identities seemed changeable, or under threat, sport and exercise could be used to reclaim folk traditions and physical expressions of national character; they could also preserve or re-create the traditional national body and its social relations. By looking back to ancient Greek or Roman traditions they linked new or reforming nations to previous great races or civilizations, and they could be obvious and public displays of cultural difference (ideally, superiority). ^10^ They could also (and perhaps this is sometimes lost in the socio-cultural considerations) claim practical medical benefits, by literally healing and strengthening the bodies of citizens.

The German system of *Turnen* and the Swedish Ling gymnastics share more common features with each other than with British competitive sports. Each is associated with one charismatic founder, Frederick Ludwig Jahn (1778–1852) or Per Henrik Ling (1776–1839), and both were inspired by the German educator Johann Friederich Guts-Muths (1759–1839), one of the first promoters of systematic physical education for children. Both schemes were also developed as systems of training and education that would rebuild degenerate national bodies. For Jahn the impetus was the Napoleonic wars, particularly the fall of Berlin in 1806, in response to which he developed a small outdoor athletics site just outside the city walls in 1811. He used his scholarly background in linguistics to choose a name for his form of body culture that, although a neologism, sounded like a traditional High German word: *Turnen* . ^11^ Ling, also a scholar of linguistics, and also interested in folklore, likewise sought to deal with a Swedish national body whose weakness had been dramatically illustrated by the loss of the northern territories (later to become Finland) to Russia in 1809. Like Jahn, his system was based on what was known of classical Greek exercise, yet the two systems were largely novel methods of training the body. In Ling’s case it was intended specifically as a scientific system, combining rational muscular training with aesthetic principles of harmony and balance. Ling’s gymnastics were also explicitly designed to improve the nation’s military strength, which is why his Royal Central Institute of Gymnastics (founded in Stockholm in 1814) was the first such European institution to get state funding. (This is also why it excluded women until 1864.) ^12^*Turnen* , by contrast, was more commonly associated with radical politics, and for this reason was banned in Prussia in 1819. It became popular again after the revolutions of 1848, maintaining its links to ideas of ‘traditional’ yet radical politics well into the twentieth century. ^13^

What sets British team and competitive sports slightly apart in this trio of Ur-sports is that they were introduced primarily to solve an educational rather than strictly military need. These sports were initially aimed not at the general population but at the upper classes and social elite, or at least their male offspring; they were therefore structured around the pedagogical and disciplinary requirements of a limited range of institutions, that is, the elite public schools and the universities of Oxford and Cambridge. This origin does not preclude a military link, and they soon became rhetorically and practically imbued with a spirit of martial imperialism. ^14^ Sports were also discussed, studied, debated and criticized, with closer focus during periods of conflict, particularly around the mid 1850s and 1900 in the wake of the Crimean and the Second South African wars respectively. ^15^ According to proponents, team sports and competitive athletics not only could improve physical robustness but could also develop discipline, self-sacrifice, leadership and stoicism in middle- and upper-class boys (and consequently in the military and political leaders of the future). ^16^

Competitive sports were also supposed to act as a distraction from other, dangerous (particularly sexual) forms of physical expression and to channel the natural violence of the male into controlled and acceptable competition. It was these games-based models of fair play, sportsmanship and muscular Christianity that reformers later wanted to extend to the bodies of the working poor to replace working-class professional and traditional sports. ^17^ The reality was that, at least after the introduction of compulsory primary education in 1870, most charitable and state-funded schools, if they provided exercise or sports at all, used *Turnen* -style drill and Ling-like gymnastics to shape the bodies of the children in their charge until well into the twentieth century (largely because of the lack of open space and other facilities, particularly in urban schools). ^18^ At the end of the century it was a French educator and anglophile Baron (Pierre) de Coubertin (1863–1937) who reinvented British sports as a thoroughly international phenomenon: having failed to introduce these activities into the French educational system, he instead resuscitated the Olympic Games, drawing as much from British sporting rhetoric as from any original Hellenic practices. ^19^

All these reinventions of sport and physical culture owe some of their popularity, or at least rising visibility, to a shared current of fear across the nations involved. Throughout the nineteenth century, military conflicts and the reality of industrialization and urbanization often highlighted the problem of a weakened citizenry, which was blamed for the flagging strength of economic and cultural institutions. Sports that promoted bodily robustness alongside traditional values spoke to these anxieties. More specifically, across Europe in the second half of the nineteenth century, these fears coalesced into a new, scientifically framed concern about progressive decline and decay, usually described as ‘degeneration’. ^20^ Degeneration is a slippery notion: it can refer to individuals, to families or to larger collectives, from nations to races; it can indicate moral decay as well as embodied physical deterioration (and often encompasses both); its aetiology can be hereditary, social, cultural or environmental; it can be an ‘urban legend’ used to give political heft or contemporary resonance to long-standing social problems. ^21^ Many scientific ideas were pressed into service as evidence for, or explanations of, degeneration, but two in particular are pervasive. One, obviously, is evolution: although often interpreted in a positivist, progressive manner, demonstrating a hierarchy of beings with (usually) the white man at the top, by the last third of the nineteenth century it could also predict gloom, decay and disaster. Progressive evolution could be halted or even reversed as bad, polluted environments or vicious lifestyles caused population to ‘de-evolve’, or revert to older, more primitive human types. Likewise, if the processes of natural selection were interfered with — if the weak, mad, bad and deviant (both individuals and races) were enabled to survive and perhaps outbreed the strong, smart and noble — then millennia of evolutionary progress could be undone.

So far there is little in this overview to explain why the dominant narrative, at least for British history, is of an antagonistic relationship between biomedicine and sport. That comes with the second scientific idea harnessed to explain degeneration: thermodynamics. That physics, and abstract physics at that, has played a role in understandings of both modernity and national decline has been a common argument since at least 1990, when Anson Rabinbach published *The Human Motor* . ^22^ The connection is as follows: the first law of thermodynamics, published in 1847 by the German physiologist Hermann von Helmholtz (1821–94) as *Über die Erhaltung der Kraft* , stated that energy could never be created or destroyed, merely changed from one form to another. This was initially an exciting promise for an industrializing continent as it suggested the possibility of limitless recyclable energy. This optimism was quickly overtaken by the consequences of the second law of thermodynamics, proposed in 1850 (and put into its complete form with the coining of the word ‘entropy’ in 1865) by the German physicist Rudolf Clausius (1822–88). Energy, Clausius argued, could not be endlessly converted, but rather became gradually changed into unusable forms, leading inevitably to the ‘heat death of the universe’: cold, dark and motionless, as all the energy in the cosmos had been converted beyond use. ^23^

This bleak fate for the macrocosm may seem very distant from day-to-day worries about fatigue and social decay, but it was absorbed into popular culture: H. G. Wells is the obvious example, as his novella *The Time Machine* (1895) includes a vision of the cold, dead universe of the future. ^24^ At a time when industrial metaphors were powerful, when nations and cities could be imagined popularly and politically as productive powerhouses ruled by the laws of energy like steam-powered engines, and when citizens were conceptualized as human machines, the question of energy conservation and energy expenditure in the microcosm was vital too. ^25^ Ample ammunition was provided for this way of thinking about the human machine by the work of a group of pioneering ‘modern’ physiologists, including Helmholtz, who explicitly linked physics to physiology, and ran a research programme in the late nineteenth century that aimed to reduce the complexity of living organisms to the mathematical simplicity of physical laws. ^26^

It is not a great leap from the human machine to Rabinbach’s ‘human motor’; framed as an energy-limited machine it was the idea (or fear) that human bodies had a fixed store of energy available to them that linked modernity, fatigue, exercise and decay. Overpressure and over-demand on the human machine would cause fatigue, damage and, eventually, progressive degeneration. This understanding worked at the micro and macro level for bodies, as well as universes; the ‘fixed store’ might be the energy of a whole person, leading to fatigue and disease if overtaxed (for example, in those who combined vigorous ‘brain work’ with physical activity), but at the same time individual muscles, organs and nerves were understood to be at risk of strain if pushed beyond ‘natural’ limits. ^27^ It is worth emphasizing that these ideas were applied less to the exhausted bodies of manual labourers than they were to the ‘nervous’ and hysterical diseases of white-collar workers, or to the wombs of women rendered infertile (or worse) by the reckless redirection of their energies into activities such as higher education or athletics. ^28^ The pervasiveness of these disease fears can be seen in the large volume of writing on diseases caused by ‘strained nerves’ or ‘stressed’ bodies, particularly the *fin de siècle* ’s most characteristic disease, neurasthenia. ^29^ Many of the suggested cures for these diseases, from expensive rest homes in the Alps to cheaper home electrical therapy kits, were explained as techniques that rejuvenated human bodies through the manipulation or replenishment of ‘energy’. ^30^

So here is an apparent paradox: at the same time as sport and physical exercise were being promoted as cures for degeneration, they were also being blamed for causing degeneration. Associations with drinking and gambling were obviously problematic, and some sports were routinely condemned as too violent or competitive to create appropriate moral feelings, but there was a connection to real bodily harm too: supporters of various competing systems of physical training often suggested that their rivals’ schemes were deficient or dangerous, either to all, or to vulnerable bodies such as women and children. ^31^ Women’s engagement in vigorous, especially outdoor or public, exercise was particularly challenging. ^32^ In some cases, then, criticisms of sport and exercise were also couched in medical and scientific terms: could too much exercise, or the wrong kind of exercise, taken at the wrong time, in the wrong way, by the wrong person, cause harm — strain or even degeneration — to minds and bodies?

These criticisms have dominated the historical landscape for at least twenty years, since the publication of *The Human Motor* (and Roberta Park and James Whorton were publishing similar arguments, although without the link to physics, in the mid 1980s ^33^ ). But more recent revisionary work has begun to paint a somewhat different picture of the decades around 1900. Both Hilary Marland and I have suggested that, while the body may have been conceptualized as a machine, it was not necessarily a fixed machine, incapable of change or improvement. ^34^ While strain was a real concern, and while energetic inputs and outputs needed to be balanced in the human machine, the capacity of bodies and muscles could be increased over time — by training. Similarly, a closer reading of what words like ‘moderate exercise’ and ‘fatigue’ actually mean in their historical context (rather than how they are understood by contemporary readers) has cast doubt on histories that describe an extremely conservative medical view of sport, with doctors condemning many forms of exercise as unhealthy. ^35^ It seems, in fact, that mainstream medical opinion was in favour of vigorous exercise by 1900, at least for the ‘normal’ person, that is, the able-bodied adult male.

## II

### MODERN STATISTICS AND MODERN STRAINS

Nonetheless, there were criticisms of sport, and although these may have been overemphasized by historians, they do provide useful opportunities to reveal the role played by sport in the construction of notions of health and well-being. While rendered invisible by their normality in many circumstances, it is ironically in situations where their normality is being debated that athletes are put, as a special category of person, into the foreground of scientific studies. That is because accusations that the athletic body is unhealthy can only be refuted by studies that are explicitly about, and specifically use, the athletic body. This is demonstrated by the fact that the first large-scale English-language scientific study of athletes was a deliberate attempt, through a statistical survey, to answer sports’ critics and prove the healthiness of vigorous (elite) sport.

In 1873 John Edward Morgan, a physician at the Manchester Royal Infirmary, published a book with a lengthy, but self-explanatory, title: *University Oars: Being a Critical Enquiry into the After Health of the Men who Rowed in the Oxford and Cambridge Boat-Race, from the Year 1829 to 1869, Based on the Personal Experience of the Rowers Themselves* . ^36^ The book was a direct response to a debate that had taken place in the letters column of *The Times* in 1867 about the possible dangers of the Oxford and Cambridge Boat Race on the Thames. This discussion was prompted by a letter from a surgeon, F. C. Skey, suggesting that the University Boat Race harmed young rowers by placing too much demand on their human machines and leading to strain, particularly heart strain. ^37^ Skey’s sceptical view of exercise has been given a disproportionate amount of attention by historians; his was a minority view, but it did provoke a range of responses (both pro- and anti-rowing and other sports), and directly inspired Morgan to start his statistical work. What became clear in the exchange of letters that followed Skey’s suggestion was that, while the debaters drew on, and tried to apply, their understanding of modern theories of fatigue and muscle function, they still relied on anecdote, personal experience and extrapolation. ^38^ There was an absence, Morgan claimed, of specific focused studies within either physiology or statistics that addressed the relationship between health and exercise. This is a salutary lesson for the historian: absence of evidence may be a way in to unspoken, tacit assumptions and taken-for-granted presumptions. One does not need to prove that sport is healthy (or safe, or moral, or fun) until there is a serious challenge to the contrary. Skey’s intervention, far from evidence of a sustained medical scepticism of sport, may be an indication of quite the opposite.

Morgan’s study fits neatly into other narratives. The nineteenth century was a period of intense mathematization, at least in western Europe, of understandings of the natural world. The laws of nature were being rendered down into predictable, mathematical, machine-like explanations. Such practices had consequences well beyond natural history or physics (or sport) as from the late eighteenth century the trend to mathematization is particularly strong in the rise of statistical studies in service to government and policing. This includes everything from new forms of economic analysis to the increasing care with which population statistics, censuses, and birth and death records were created and collected. ^39^ Part of what makes modern sports ‘modern’, at least according to Allen Guttmann, is that it participates in this move to quantification: towards ‘countable’ sports and feats that can be measured, specifically, by time, distance or weight. ^40^ Morgan was therefore drawing on contemporary trends in study and evidence when he sent out surveys to ex-rowers, their friends and families, and rowing clubs. He also relied on the new statistics himself, seeking out life tables and mortality statistics (usually collated and provided by insurance companies) to give estimates of ‘average’ or ‘normal’ death and sickness rates to act as a control against the data he collected from and about rowers. His conclusion was unambiguous: varsity rowers lived at least as long as their non-rowing peers, and in general experienced no negative health effects for having rowed in the Boat Race.

Morgan’s work was widely read, and influential: at least a few doctors and cardiologists who had been in the minority, criticizing sport, revised their position after the publication of *University Oars* . ^41^ That is, it was widely read and influential in British intellectual circles, while it seems to have had less impact elsewhere in Europe, where similar patterns of sceptical challenge and statistical rebuttal can be seen in medical works even after Morgan’s publication. To take one example, the German doctor George Kolb published an equally long-windedly titled book in 1887 (fourteen years after Morgan’s), which was translated into English as *Physiology of Sport: Contributions towards the Physiology of a Maximum of Muscular Exertion, especially Modern Sports, as Rowing, Athletics, Gymnastics, Cycling, Swimming, etc.*^42^ In the introduction to this text Kolb claimed that he ‘expect[ed] to find’ that the professional sportsman was an ‘invalid’. ^43^ Given Kolb’s own enthusiasm for (and participation in) sports, his suggestion that he genuinely thought he would find so much disability linked to sport reads something like hyperbole; his conclusion is the opposite, that sport is healthy and safe. We should read his introduction in light of his conclusion, since data that converts an author may consequently seem more convincing to a reader. In any case, it can have been no disappointment to Kolb to find that, far from experiencing heart strain, ‘scarcely *one single man* was without an entirely normal and healthy circulation of the blood’. ^44^ Overall, Kolb concluded that, while there were some risks in sport, mostly of the traumatic and accidental kind, the moral and medical benefits on average outweighed any risks. Moreover, as he had chosen to study bodies that engaged in the most extreme forms of exercise, it was logical to assume that more moderate forms of sport would have even fewer potentially negative outcomes.

*Physiology of Sport* does not offer us any particularly novel conclusions, but it is widely referenced and cited nonetheless: this is probably because of its unfortunate title in translation. Historians (and even a contemporary reviewer in the *British Medical Journal* ) refer to it as one of the first texts on exercise physiology. ^45^ As such, it is a disappointment, as the review makes clear, criticizing not only Kolb’s translation efforts, but also the balance between data and analysis. ^46^ This is unfair, as Kolb’s purpose was not to write a textbook on exercise physiology. *Physiology of Sport* was instead an analysis of the effects of sport on health and well-being, which is why so much of the book is given over to clinical and statistical observations: measurements of athletes, analyses of their urine, records of their pulse rates and heart sizes, and so on. Although this is bulked out with a discussion of the nature of fatigue — which for Kolb is a purely biochemical phenomenon caused by the accumulation of ‘fatigue stuffs’, a view rather ahead of its time — the book is therefore not a work on exercise physiology as the modern (or contemporary) reader might imagine it. Taking Kolb’s book as the first on exercise physiology certainly shores up arguments about modern science and modern sports in the nineteenth century: how odd it looks if the first book on exercise physiology is so focused on the debates over the health of sport that it barely contains any exercise physiology at all. Of course, that absence looks less indicative when we read Kolb’s book as a survey work like Morgan’s, and not an attempt at a textbook at all.

Victorians wanting a primer on exercise physiology would have been better off reading French rather than German literature, as a stronger candidate for the first book on this topic was written by the physiologist Fernand Lagrange (1845–1909): *Physiologie des exercices du corps* , published in 1889, with an English translation in 1898. ^47^ Even here it is apparent that it is conflict over sport and exercise that renders sporting bodies visible as objects of scientific (or, in this case, state) scrutiny: normality and health were part of a high-level debate that was far more intense and at least as divided in France as it was in Britain (or Germany). Lagrange was one of a growing body of physiologists and doctors in France who were heavily involved with the reform of physical education and sport, a phenomenon better studied in the twentieth century, but with clear roots in the late nineteenth. ^48^ France follows the pattern we have already seen in Germany, Sweden and Britain: reforms relating to exercise and physical culture arose in part as reactions to fears of national decline and degeneration, particularly after the Franco-Prussian War of 1870–1. The immediate response to military defeat was to institute military drill and training in state schools, but throughout the 1880s and 1890s a hygienist movement argued the case for more general health and education, suggesting that an already healthy citizen could more rapidly be trained in the specialized skills of the military when necessary.

Western scientists, doctors and other middle-class professionals became imbricated in government throughout the nineteenth century, often by making appeals to their expertise in pressure points between the citizen and the state: public health, education, economics, industrial research, military strength. Physical exercise and sport offered an obvious way for biomedical and physiological professionals to become involved in government reform and intervention. Lagrange was one such, appointed to the head of a commission charged with reforming gymnastics, serving alongside the physiologists and film pioneers Georges Demenÿ (1850–1917) and Étienne-Jules Marey (1830–1904). ^49^ Opinions on sport and exercise in late nineteenth-century France were clearly split. In 1888 the Ligue Girondine d’Éducation Physique was founded by the physician Philippe Tissié (1852–1935), and it promoted the use of gymnastics and similar forms of exercise, setting them up in explicit contrast to competitive and ‘violent’ team games and track sports. These were, of course, games of exactly the sort that were championed by Baron de Coubertin, although he framed them as manly and improving, rather than violent. Perhaps unsurprisingly then, when Tissié studied elite and competitive athletes from team and track sports, his investigations tended to suggest that such high-level sport was, after all, unhealthy. Tissié’s views therefore reflected, and possibly led, those of the Ligue, but need to be read as being anti-British (or at least anti French Anglophile) as much as they were anti competitive sports.

Despite this political complication, it is Tissié and not Lagrange who is usually cited as the pioneer of French sports medicine, not least because of the emphasis on him in John Hoberman’s seminal work on the machine metaphor in sport, *Mortal Engines* . ^50^ Certainly Tissié’s work resonates well with the concerns of twentieth- and twenty-first-century sport; he is best remembered for his studies of drugs and stimulants, particularly his series of studies of the endurance cyclist Stéphane, which, of all nineteenth-century experiments involving an athlete, historians must have examined the most closely. ^51^ As this article demonstrates, however, this focus is misleading. Tissié’s negative attitude towards the healthiness of elite and endurance sports was not representative of a hegemonic medical view, and while he may have been one of the first physiologists to study athletes with the assumption that they were abnormal (and possibly unhealthy), he was far from the first to study them per se. Lagrange, whose work is more general and more representative, was generally in favour of competitive sports and athletics. Although he does suggest that extreme exercise can cause strain, distress and heart disease, in doing so he makes reference to particularly atypical sports; for example, he says, ‘Professional runners, some of whom in Africa traverse almost incredible distances, are in the end usually affected with passive dilatation of the heart, resulting from exhaustion of the organ’. ^52^ He was sceptical about more moderate running as a healthy form of exercise too, as it so often led to breathlessness, fatigue and collapse; but again, even here he cannot be recruited into the argument that doctors routinely opposed sport, as he suggests that part of the problem was that in France, unlike in England, sports coaches were not available. ^53^ He specifically emphasizes the fact that bodies could be conditioned into safe participation at a level of performance unavailable to the untrained body, and he therefore fits better with the more positive reading of medicine and sport indicated by more recent historical work. ^54^

One other intriguing aspect of Lagrange’s work is that he makes no distinction between exercise in sport and exercise in manual labour:

Scientifically speaking there is no difference between the professional labour which circumstances demand from the peasant or workman, and the more or less refined exercise to which a sportsman devotes himself.

The difference lies in the participants’ way of life:

The gentleman has his exercise at his own hours, regulates to his own taste the time he allots to it, following the calls of hygiene, diet and rest, while the poor man works too much, feeds badly, and sleeps little. This is why work wears out the one, while exercise strengthens the other. ^55^

Even in the late nineteenth century it was not obvious to researchers that the ‘athlete’ was a useful ‘natural kind’, that is, a useful category of human being for specific and special analysis, or an important category for scientific, medical or statistical study. It was not until the early twentieth century that the bodies of athletes (now including women) were reconceptualized as something other than normal; in other words, that these were bodies that were not subject to the normal laws of physiology, or which might need special training and treatment. ^56^ Prior to this point it may not have been important, or apparently relevant, to mention that a human guinea pig rowed for his university, or was a keen physical culturalist, taking cold baths and long runs before breakfast. What this means is that the use of athletes and sporting bodies to generate knowledge and facts about the world is often effaced. The next section reclaims one of these human guinea pigs by reconsidering Edward Weston’s story. Significant not only in its own right as part of a two-generation international debate, the walk in the Royal Agricultural Hall also matters because of the clear agency of the athlete involved; it is an indicative window into the invisible participants of scientific work.

## III

### FUELLING THE HUMAN MACHINE

Nations concerned with the degeneration and ‘thermodynamics’ of their populations were as interested in fuel as they were in fatigue; that is, how should the human machine be fed, and how did that food relate to their industrial or economic output? The question of the source of bodily energy was an active debate throughout the nineteenth century, and was at its core a deceptively simple physiological question: what was the source of muscular and nervous energy, and how was it replenished? In 1842 the German chemist Justus Liebig (later Justus von Liebig; 1803–73) put forward the suggestion that it was protein that was the bodily, and therefore dietary, source of energy for movement. ^57^ In Liebig’s model the muscles of the body were literally broken down through a chemical reaction that liberated energy, which was then converted into movement and heat. After exercise the muscles were rebuilt during rest periods using protein from the diet. This was not just an abstract laboratory theory; it also resonated strongly with the lived experience of high-performance and professional sports people, as high-protein diets were consistently favoured by practitioners and recommended in training books and biographies. Evidence for Liebig’s theory could also be drawn from experiment owing to the presence of urea (a waste product from protein metabolism) in the urine of people doing intense work, and coincided with common experience as the strongest and most fatigue-resistant members of the human race tended to be those with good musculature (which, in Liebig’s system, meant they had a larger store of energy to draw from).

With sporting superstition, ‘common sense’ and laboratory evidence all in sync, it is hardly surprising that the idea that protein is essential to exercise remains, even into the twenty-first century. ^58^ But it was not long before Liebig’s theory was challenged. In 1860 Carl von Voit (1831–1908), originally hired as an assistant physiologist in Liebig’s research group, produced some ambivalent results using dogs on treadmills. ^59^ In 1866 Voit and the Bavarian physiologist Max Joseph von Pettenkofer (1818–1901) were given 2,800 guilders by the Bavarian royal family for the construction of a human-sized respiratory chamber to extend this work. ^60^ This sealed room, large enough to hold a human being with space to move around, allowed the experimenters to calculate and measure every input and output: gases, solids, liquids, and the heat and movement generated by their subject. Their conclusions were cautious, possibly because Liebig was still such a powerful and idolized character, so, although their figures strongly suggested that bodily energy drew on sources other than dietary protein, they maintained that protein was nonetheless the main source of energy in human beings. ^61^ (Voit went on to set what became known as the ‘Voit standards’, daily minimum recommended amounts of protein for working and sedentary men. Although these were used in the design of dietaries by private and state-run institutions, and social reformers, across Europe, at 118 grams per day for the labouring man they were considered mistakenly high by the early twentieth century.) ^62^

It took two bolder, self-experimenting physiologists really to challenge Liebig’s ideas. In August 1865 the physiologist Adolf Eugen Fick (1829–1901) and the chemist Johannes Wislicenus (1835–1902) picked the Faulhorn as the ‘laboratory’ for an elegantly simple experiment to test Liebig’s theory. The Faulhorn was a popular destination for scientific researchers in the mid and late nineteenth century, not least because it conveniently had a hotel on the summit. ^63^ Assuming that Liebig’s theory was correct, an ascent of the mountain would be powered only by the breakdown of protein in the climbers’ muscles, which could be quantified by measuring the amount of urea in their urine. To avoid any contamination by dietary protein, Fick and Wislicenus relied on carbohydrates as climbing food, eating fried starch paste and drinking sugary tea on the ascent. The climb took eight hours, and afterwards they performed a fairly simple set of calculations to estimate the energy required to lift their body weights the height of the Faulhorn. Of course, these calculations themselves drew from work in thermodynamics, and were part of the practices that rendered the human body into a machine whose inputs and outputs could be precisely measured, and thus understood. The results were unambiguous: the breakdown of muscle, as indicated by their urea production, was not sufficient to account for the energy they used in the climb. ^64^ The disparities were not huge (250 calories released versus 305 burned for Fick, and 249 versus 352 for the slightly heavier Wislicenus), but the researchers had made no allowance for any other energy needs: basal metabolic functions (digesting that fried paste), taking a slightly meandering path up the mountain, and so on. So the number of calories calculated as having been used was a minimum, and the difference between the energy available from protein metabolism and the energy burned even greater. Something had to make up that shortfall. It was therefore clear, they argued, that at least some bodily energy must be provided by the non-nitrogenous parts of the diet; in other words, fats and sugars also played a role in fuelling the moving body.

Were this a traditional history of diet or science, my account would understand Fick and Wislicenus’s research as the key disproof of Liebig’s protein theory of energy, and would move on to the rather long story of how scientists discovered the exact roles of fats and carbohydrates, culminating in 1922 in the Nobel Prize in physiology or medicine for the British physiologist A. V. Hill (1886–1977) and the German biochemist Otto Meyerhof (1884–1951) for their work on the detailed metabolism of muscles. But such accounts ignore the fact that this debate was far from settled in the nineteenth century. For doctors, scientists and athletes the relationship between the contents of the diet and the output of the body was not yet certain. As late as 1878 the prominent American physiologist Austin Flint Jr (1836–1915) was defending Liebig’s protein hypothesis and, more significantly for this story, doing so by using athletes as his experimental subjects. In a lengthy article published in the *Journal of Anatomy and Physiology* in 1877, Flint summarized the state of the field, and heavily criticized the conclusions of Voit, Fick and Wislicenus, and particularly the work of the British physiologist Frederick William Pavy (1829–1911). ^65^ Flint’s original research work, undertaken in the early 1870s, followed a familiar pattern: subjects performed variable amounts of exercise, their food intake was observed or controlled, and their urine collected and analysed for traces of urea. Again, this is a pattern of thermodynamically informed research, looking at inputs and outputs. What was more unusual in Flint’s work was his use of sportsmen, particularly the long-distance pedestrian Edward Weston, to prove his theory that heavy exercise increased excretion of the waste products of protein metabolism (which implied that muscles were actively consumed in the process of exercise). The consequence of Flint’s choice was that Weston, and more particularly his body and bodily fluids, became key players in a series of acrimonious scientific debates.

When sporting and financial motives drew Weston to the United Kingdom in 1876, he was immediately courted by Flint’s nemesis, Pavy. The latter was then a practising doctor and lecturer in physiology at Guy’s Hospital in London. By the 1870s he had gained a reputation as a notable researcher, a prolific publisher of experimental studies and a founder member of the Physiological Society. He also had a tendency to tackle, and directly challenge, the European ‘greats’ of experimental physiology. His most recent major set of works had been an attempt to overthrow ideas about sugar metabolism developed by the ‘father of physiology’, Claude Bernard (1813–78). National rivalry, as often intellectual as military, is clearly a theme in this story; in this case, European continental physiology had taken an experimental (and physics-oriented) turn in the second half of the nineteenth century, and, while many in Britain criticized this new direction, just as many saw it as proof of the stagnation of British physiology (and possibly all forms of British science). ^66^ Weston’s transatlantic trip offered Pavy an opportunity far too good to miss: not only could he challenge a great German scientist’s ideas, but he could also tackle one of his own personal critics using the very same human guinea pig.

Weston, presumably used to the odd demands of physiologists by this point, agreed to collect all the urine he passed during his attempt to walk 115 miles in twenty-four hours (he actually completed ‘only’ 109.5 miles). Unfortunately, this sample was ruined as ‘through inadvertence on the part of one of the attendants, slops were thrown into [the urine bucket] and it was rendered useless for analysis’. ^67^ This mistake turned out to be a lucky one, as we shall see, but for his first article on diet and sport Pavy had to use a post-race urine sample, and the urine collected from Weston’s only challenger, the British walker Mr Perkins. (Mr Perkins’s ‘thin slipper shoes’ did him no favours and foot pain forced him to switch to canvas lace-up boots before dropping out after 65.5 miles and 14 hours 30 minutes.) ^68^ Weston also agreed to save his urine during a subsequent forty-eight-hour 180-mile walk, and over two long articles in *The Lancet* Pavy published his analyses of Weston’s dietary intake and performance and scrutinized, and published pictures of, his urine. ^69^

Pavy’s conclusions were couched explicitly in terms informed by the new science of thermodynamics:

It is now an established doctrine that force, like matter, can be neither created nor destroyed. The different forms of force are mutually convertible the one into the other . . . What is true of force in the inorganic world is equally applicable in the organic. The force manifested by living beings has its source by transmutation from other forms which have pre-existed. The food of animals contains force in a latent state. Properly regarded, food must be looked upon, not simply as so much ponderable matter, but as matter holding locked-up force. ^70^

From this analysis, this calculation of matter in and force out, Pavy was able to correct, if not outright contradict, Liebig’s theory about the unique centrality of protein to the human motor. Pavy concluded that protein alone could not have powered Weston’s walk, and that fats and carbohydrates also played a part in fuelling the human body. This confirmed Fick and Wislicenus’s original finding, although the two Germans had already gone further, arguing that exercise had no effect on protein metabolism at all (that is, it did not stimulate the breaking down of muscles). Pavy could not go that far with the data to hand, and concluded that doing hard or very hard exercise did increase the body’s need for protein, and could cause muscle to be digested, but he also made a strong case that protein should be dethroned as the main or only source of bodily energy.

Pavy’s analysis of Weston’s bodily fluids almost immediately became part of a controversy, but not one that challenged his understanding of human fuel. Instead the problem was that a British public health specialist levelled a charge of doping against Weston. J. Ashburton Thompson (1846–1915), a keen epidemiologist and public health specialist, had been following Weston’s feats and publishing his own independent physiological observations on Weston’s races. ^71^ Thomson’s dispute with Pavy revolved around the use of coca. Weston was sometimes in the habit of chewing the leaves of the coca plant ( *Erythroxylum coca* ) and Thompson pointed out that this would materially affect the outcome of urine studies. Coca, he said, affected the production of urine and ‘retard[ed] the waste of tissues’ (that is, it prevented muscle breakdown), and would therefore compromise the results of Pavy’s otherwise meticulous study. ^72^ As luck would have it, the race in which Thompson had seen Weston chewing coca was the same race in which there had been a disaster with the urine bucket (this was the one contaminated with ‘slops’ and discarded). This happy accident rescued Pavy’s scientific claims as Weston insisted that this was the only time he had used coca, so all the other urine samples were untainted and the analysis stood. ^73^

This is, to my knowledge, the first recorded incident of a complaint about doping in modern sports. At a basic level it is interesting because it is a useful corrective to the more celebratory narratives of British nineteenth-century sport that bring to the fore an amateur ethos, which is read as one that did not allow athletes to train, to take drugs or stimulants, or to ‘try too hard’ in any way (and certainly not to take part in serious academic study of sport). That the complaint is not about the use of a stimulant per se, but about its consequences for scientific work, is a salutary reminder that absences are sometimes poor guides to the lived reality of the past. A lack of complaints about doping can either indicate that athletes avoided pharmaceutical assistance or, more likely (as this case shows), demonstrate a very different attitude towards the rights and wrongs of doing so. This doping accusation also opens up a second, far more important absence: no one, after all, suggested that an elite athlete capable of performances at world-champion level was perhaps a pretty poor model for understanding the activity of the normal human body. At a time when population statistics was establishing itself as a vital political and social science, questions of norms, averages and fair comparisons were being discussed in many similar situations, so why not here? ^74^ Again, the absence is leading: there are few occasions when studies on athletes were questioned in this way, and these relate almost entirely to cases in which there is a pointed political issue at stake, most commonly the feeding of prisoners and workhouse inmates.

Amid the nineteenth-century anxiety about healthy and unhealthy populations is an ongoing set of conflicts about the diets of captive or institutionalized populations: prisons, hospitals, workhouses, military bases and, later in the century, state-funded schools. In Britain, in particular, the fervent and often medical-led opposition to the poor law reforms of the 1840s pointed specifically to the alleged inadequacy of diets in the workhouses, and there were very public clashes between reformers, politicians and epidemiologists over issues of starvation and poverty. ^75^ It therefore mattered intensely, for political, economic and ethical reasons, whether man could live by bread alone, or if generous portions of ‘animal food’ were also needed for normal physical work. ^76^ Physiological studies of nutrition were therefore politically loaded: as well as being a locus for international rivalry, they were used by reformers (and their opponents) as evidence of the correct or incorrect dietary regime for state-run institutions. Probably the most famous work was that of Edward Smith (1819–74), who studied the diets of prison and workhouse inmates, particularly prisoners who had to work on the treadmill at Brixton prison, and attempted to come up with ‘scientific’ dietaries. ^77^ Smith’s work was controversial, but he did succeed in leading and influencing government inquiries into the diets of the poor and the incarcerated. ^78^ Specifically, he also inspired Fick and Wislicenus to climb the Faulhorn after he criticized Liebig’s theory that proteins supplied bodily energy. ^79^ In turn, Fick and Wislicenus inspired the military doctor Edmund Parkes (1819–76) to reconsider diets in the British army. Parkes, professor of military hygiene in the Army Medical School and of clinical medicine at University College London, used exercising soldiers as subjects of study in the mid 1860s to confirm Fick and Wislicenus’s findings. ^80^ This work had direct consequences in the reconstruction of army dietaries and ration packs. ^81^ In turn, Parkes’s work, like Pavy’s, was taken up and criticized by Flint when he studied Weston in the United States, so this becomes a transatlantic as well as a European story, involving soldiers, prisoners, social reformers, scientists and athletes.

Pavy’s work on athletes did not lead directly to a revolution in the sporting diet; in fact, he is best remembered as a world expert in the construction of diets for the treatment of diabetes. ^82^ (At this time the only treatment for diabetes was a drastically reduced diet, eliminating as much carbohydrate as possible.) But his research did feed into subsequent work on the diets of soldiers, prisoners and the poor. So it is worth reiterating that even in this extremely febrile research field, with relatively high political stakes, it was extremely rare for studies to be criticized because they used athletes as subjects. When they were criticized, it was almost exclusively because of the type of work done; that is, it was not clear that climbing a mountain or running were equivalent forms of labour to the treadmill or rock breaking (though, as we have seen from Kolb’s book on the physiology of sport, the equivalence of forms of labour became more widely acknowledged towards the end of the century). Athletic bodies, therefore, could still be representative of normal human function. Indeed, when the American chemist Wilbur Atwater (1844 –1907), with his assistant Charles Langworthy, published an extensive review of the existing research literature on nutrition and diet in 1898, he divided up the 3,800 studies he identified into those studying healthy or unhealthy bodies, in normal or abnormal situations, but he took no account of whether the subjects were elite athletes or sedentary bank clerks. ^83^

This habit of assuming that the athletic body is the normal body, especially as it relates to politically applicable research, continued into the twentieth century. Perhaps the most egregious (and possibly last) example was the use of the champion marathon runner Clarence DeMar by the Harvard Fatigue Laboratory in the 1920s and 1930s. A joint venture between the Harvard Medical School and Harvard Business School, the laboratory had a clear founding aim to study the problems of labour (with a view to easing the problems of management). The fatigue of working men, and the implied need, if any, for the reform of working hours, was one such puzzle they tackled, and one that researchers sometimes examined by using the performance of elite sportsmen rather than that of ordinary factory-workers. ^84^

## IV

### CONCLUSION

The first encounters between modern biomedical science and modern sport helped to codify sport as a healthy male activity, provided a space for discussions about national character and national scientific standing, and even created a site for the generation of ‘evidence-based’ policy making — at least when it came to designing military rations, feeding workhouse inmates or designing compulsory physical education courses for state schools. ^85^ That we do not know much about these encounters is the result of two forms of invisibility: one a consequence of nineteenth-century assumptions about bodies, and the other a consequence of historians’ assumptions about the nineteenth century.

Pavy is an excellent example of the way in which blind spots are generated by our historiography. Unfortunately for his long-term reputation he was neither so wrong about metabolism that he can appear as an antagonist (or as evidence of the backwardness of British physiology), nor so right that he can be celebrated as a pioneer or visionary hero. Indeed, he is difficult to write into histories of science because, despite being a keen provocateur taking on the ‘big names’, he is a deeply inconvenient historical actor who entirely fails either to demonstrate or to contradict any of the current trends or arguments about science in the nineteenth century. Key to the existing historiography is the rise of the laboratory as a crucial site of authority and knowledge production in the European life sciences. This is a story of national differences, as mentioned above, as in continental Europe, particularly in Germany and France, the institutions and facilities for laboratory-based experimental physiology appeared considerably before equivalent institutions in the United Kingdom or the United States. This led to debates about whether this new approach was either correct and modern, or reductive and simplistic, and whether British science was wise or foolish to neglect it. ^86^

Pavy certainly took part in these debates. Having trained with Claude Bernard in France, he complained of ‘the stagnancy of English physiology which was kept afloat by amateurs like [myself] in whatever time they could spare from private practice’. ^87^ On the other hand, one of his major criticisms of Liebig was the chemist’s reliance on oversimplified laboratory models and an over-reductive mathematical theory of biology, which could not, in Pavy’s view, accurately represent the complexity of living systems. Understanding these two views makes sense of Pavy’s approach to experiment: he was clearly committed to clinical experimental physiology, doing field studies and using whole organisms at the same time as running detailed laboratory studies with a microscope and analytical chemical equipment. He used much quantification and was well informed by theoretical and thermodynamic models, but also used his experience of sports people and patients, and ‘common knowledge’ (for example, about athletes’ preferred diets) in his scientific publications. Neither fish nor fowl, he is not useful in arguing or illustrating either side of a historiographic debate, if it can only be represented as polarized.

This is somewhat unfortunate as it is these mixed-site, field-based and whole-body or clinical kinds of physiology that had the clearest application to crucial political and social problems, and this is the site where sport and science most often meet. Ignoring studies of these kinds in favour of telling stories about the rise of the laboratory, or about cross-Channel debates on the philosophy of experiment, has made it less likely that we would be able to spot the athletic body when it is engaged in scientific work. Weston’s story is not just about a meeting of science and sport that goes against the usual accounts of absence or antagonism, but also one in which the agency of the sportsman can be properly recognized. ‘Mr Weston enters with as much enthusiasm into the spirit of these researches as into his walk, and has placed every facility at my disposal’, wrote Pavy. ‘It is only a just tribute to him to say that science is indebted to him for his desire to aid its advance’. ^88^ Without stories like this, athletes disappear into the scientific and medical literature; as ‘normal’ human beings the amateur athlete or professional sportsman is rarely flagged in publications. Consequently, hundreds of athletes have almost certainly participated in human experiments and studies only to be anonymized as subjects identified only with an initial, a gender, an age. They only become visible when the work is a study of sport as a potential problem (such as Kolb or Morgan’s surveys), which has inevitably skewed our reading of past medical encounters with sports, as well as hiding the agency and identity of the participants in scientific work.

The fact that nineteenth-century writers on sport and science thought that the active male body was normal, the standard body for scientific work, or something to take for granted, does not mean we should, as historians, agree with them. ^89^ Weston’s long walk in the Royal Agricultural Hall in 1876 is an opportune historical moment for intervention and analysis. Not only does it allow a revisionist conversation about the relationship between modern sport and modern science, but it also requires that those dialogues should be properly placed in the context of a series of other narratives: about body culture and national identity; about international relationships and fears of social, cultural and physical degeneration; about the history of welfare and social policy; about an understanding of societies and nations that rendered citizens as energetic machines. None of these narratives can be fully fleshed out in this article, but it does serve to demonstrate a useful place where a historian can step in and open out the ramifications of a previously under-considered relationship. It should remind us of the analytic power of absences.

## Footnotes

^1^ F. W. Pavy, ‘The Effect of Prolonged Muscular Exercise on the System, [Part I]’, *Lancet* , cvii, 2739 (1876).

^2^ Tony Collins, *Sport in Capitalist Society: A Short History* (London, 2013); Nick Harris, Helen Harris and Paul Marshall, *A Man in a Hurry: The Extraordinary Life and Times of Edward Payson Weston, the World’s Greatest Walker* (London, 2012); Wray Vamplew, *Pay Up and Play the Game: Professional Sport in Britain, 1875–1914* (Cambridge, 1988); Richard Holt, *Sport and Society in Modern France* (London, 1981).

^3^ David Aubin, ‘The Hotel that Became an Observatory: Mount Faulhorn as Singularity, Microcosm, and Macro-Tool’, *Science in Context* , xxii (2009).

^4^ Roberta J. Park, ‘Physicians, Scientists, Exercise and Athletics in Britain and America from the 1867 Boat Race to the Four-Minute Mile’, *Sport in History* , xxxi (2011); Neil Carter, ‘Metatarsals and Magic Sponges: English Football and the Development of Sports Medicine’, *Journal of Sport History* , xxxiv, 1 (2007); Roberta J. Park, ‘ “Mended or Ended?” Football Injuries and the British and American Medical Press, 1870–1910’, *International Journal of the History of Sport* , xviii (2001); R. J. Park, ‘High-Protein Diets, “Damaged Hearts”, and Rowing Men: Antecedents of Modern Sports Medicine and Exercise Science, 1867–1928’, *Exercise and Sports Sciences Reviews* , xxv (1997); James C. Whorton, ‘ “Athlete’s Heart”: The Medical Debate over Athleticism, 1870–1920’, *Journal of Sport History* , ix, 1 (1982).

^5^ Wray Vamplew, ‘Sport and Industrialization: An Economic Interpretation of the Changes in Popular Sport in Nineteenth-Century England’, in J. A. Mangan (ed.), *Pleasure, Profit, Proselytism: British Culture and Sport at Home and Abroad, 1700–1914* (London, 1988).

^6^ See n. 4.

^7^ Timothy Lenoir, *Instituting Science: The Cultural Production of Scientific Disciplines* (Stanford, 1997); Arleen Marcia Tuchman, *Science, Medicine, and the State in Germany: The Case of Baden, 1815–1871* (New York, 1993); Andrew Cunningham and Perry Williams (eds.), *The Laboratory Revolution in Medicine* (Cambridge, 1992); John E. Lesch, *Science and Medicine in France: The Emergence of Experimental Physiology, 1790–1855* (Cambridge, Mass., 1984).

^8^ Most of these are listed in Park, ‘Physicians, Scientists, Exercise and Athletics in Britain and America from the 1867 Boat Race to the Four-Minute Mile’.

^9^ For a specific re-reading of some of these terms, see Vanessa Heggie, ‘A Century of Cardiomythology: Exercise and the Heart, *c.* 1880–1980’, *Social History of Medicine* , xxiii (2010).

^10^ Felix Saure, ‘Beautiful Bodies, Exercising Warriors and Original Peoples: Sports, Greek Antiquity and National Identity from Winckelmann to “Turnvater Jahn” ’, *German History* , xxvii (2009).

^11^ Michael Krüger, ‘Sports in the German University from about 1900 until the Early Years of the Federal Republic of Germany: The Example of Muenster and the “Westfaelische Wilhelms-University” ’, *International Journal of the History of Sport* , xxix (2012).

^12^ Anders Ottosson, ‘The First Historical Movements of Kinesiology: Scientification in the Borderline between Physical Culture and Medicine around 1850’, *International Journal of the History of Sport* , xxvii (2010).

^13^ Christiane Eisenberg, ‘Charismatic Nationalist Leader: Turnvater Jahn’, in Richard Holt, J. A. Mangan and Pierre Lanfranchi (eds.), *European Heroes: Myth, Identity, Sport* (London, 1996), 14–20.

^14^ Tony Mason and Eliza Riedi, *Sport and the Military: The British Armed Forces, 1880–1960* (Cambridge, 2010); J. A. Mangan, *Athleticism in the Victorian and Edwardian Public School: The Emergence and Consolidation of an Educational Ideology* , rev. edn (London, 2000); J. A. Mangan, ‘ “Muscular, Militaristic and Manly”: The British Middle-Class Hero as Moral Messenger’, *International Journal of the History of Sport* , xiii (1996).

^15^ Vanessa Heggie, ‘Lies, Damn Lies, and Manchester’s Recruiting Statistics: Degeneration as an “Urban Legend” in Victorian and Edwardian Britain’, *Journal of the History of Medicine and Allied Sciences* , lxiii (2008).

^16^ Bruce Haley, *The Healthy Body and Victorian Culture* (Cambridge, Mass., 1978); H. S. Ndee, ‘Public Schools in Britain in the Nineteenth Century: The Emergence of Team Games and the Development of the Educational Ideology of Athleticism’, *International Journal of the History of Sport* , xxvii (2010).

^17^ Peter Bailey, *Leisure and Class in Victorian England: Rational Recreation and the Contest for Control, 1830–1885* (London, 1978); Neal Garnham, ‘Both Praying and Playing: “Muscular Christianity” and the YMCA in North-East County Durham’, *Journal of Social History* , xxxv (2001).

^18^ John Welshman, ‘Physical Culture and Sport in Schools in England and Wales, 1900–40’, *International Journal of the History of Sport* , xv (1998); H. S. Ndee, ‘Physical Education in State and Private Schools in Britain in the Late-Nineteenth and Early-Twentieth Centuries: Elementary Schools and Other Schools’, *International Journal of the History of Sport* , xxvii (2010).

^19^ Douglas Brown, ‘Modern Sport, Modernism and the Cultural Manifesto: De Coubertin’s *Revue Olympique* ’, *International Journal of the History of Sport* , xviii (2001).

^20^ J. Edward Chamberlain and Sander L. Gilman (eds.), *Degeneration: The Dark Side of Progress* (New York, 1985); Daniel Pick, *Faces of Degeneration: A European Disorder, c.1848–c.1918* (Cambridge, 1989); Heidi Rimke and Alan Hunt, ‘From Sinners to Degenerates: The Medicalization of Morality in the Nineteenth Century’, *History of the Human Sciences* , xv (2002).

^21^ Heggie, ‘Lies, Damn Lies, and Manchester’s Recruiting Statistics’.

^22^ Anson Rabinbach, *The Human Motor: Energy, Fatigue, and the Origins of Modernity* (New York, 1990).

^23^ Iwan Rhys Morus, *When Physics Became King* (Chicago, 2005).

^24^ H. G. Wells, *The Time Machine* (London, 1895).

^25^ Jennifer Karns Alexander, *The Mantra of Efficiency: From Waterwheel to Social Control* (Baltimore, 2008); Iwan Rhys Morus, ‘The Measure of Man: Technologizing the Victorian Body’, *History of Science* , xxxvii (1999); George Rosen, ‘The Conservation of Energy and the Study of Metabolism’, in Chandler McC. Brooks and Paul F. Cranefield (eds.), *The Historical Development of Physiological Thought: A Symposium Held at the State University of New York Downstate Medical Center* (New York, 1959).

^26^ Lenoir, *Instituting Science* .

^27^ Chamberlain and Gilman (eds.), *Degeneration* ; Pick, *Faces of Degeneration* ; Rimke and Hunt, ‘From Sinners to Degenerates’; Wells, *Time Machine* ; Rabinbach, *Human Motor* ; Kelly Hurley, ‘Hereditary Taint and Cultural Contagion: The Social Etiology of Fin-de-Siècle Degeneration Theory’, *Nineteenth-Century Contexts* , xiv (1990); Charles E. Rosenberg, ‘Pathologies of Progress: The Idea of Civilization as Risk’, *Bulletin of the History of Medicine* , lxxii, 4 (1998).

^28^ Patricia A. Vertinsky, *The Eternally Wounded Woman: Women, Doctors, and Exercise in the Late Nineteenth Century* (Manchester, 1990).

^29^ Roberta J. Park, ‘ “Soldiers May Fall but Athletes Never!” Sport as an Antidote to Nervous Diseases and National Decline in America, 1865–1905’, *International Journal of the History of Sport* , xxix (2012); Barbara Sicherman, ‘The Uses of a Diagnosis: Doctors, Patients, and Neurasthenia’, *Journal of the History of Medicine and Allied Sciences* , xxxii (1977).

^30^ Paola Bertucci and Giuliano Pancaldi, *Electric Bodies: Episodes in the History of Medical Electricity* (Bologna, 2001).

^31^ Pål Augestad, ‘Architecture and the Education of the Body: The Gymnasium in Norwegian Physical Training, 1889–1930’, *International Journal of the History of Sport* , xx (2003).

^32^ Anaïs Bohuon and Antoine Luciani, ‘Biomedical Discourse on Women’s Physical Education and Sport in France, 1880–1922’, *International Journal of the History of Sport* , xxvi (2009); Kathleen E. McCrone, ‘ “Play Up! Play Up! And Play the Game!” Sport at the Late Victorian Girls’ Public School’, *Journal of British Studies* , xxiii (1984); Roberta J. Park, ‘ “Embodied Selves”: The Rise and Development of Concern for Physical Education, Active Games and Recreation for American Women, 1776–1865’, *Journal of Sport History* , v, 2 (1978).

^33^ Roberta J. Park, ‘Health, Exercise, and the Biomedical Impulse, 1870–1914’, *Research Quarterly for Exercise and Sport* , lxi (1990); Whorton, ‘Athlete’s Heart’.

^34^ Hilary Marland, *Health and Girlhood in Britain, 1874–1920* (Basingstoke, 2013); Heggie, ‘Century of Cardiomythology’.

^35^ See Heggie, ‘Century of Cardiomythology’.

^36^ J. E. Morgan, *University Oars* (London, 1873).

^37^ F. C. Skey, ‘Athletics’, *Times* , 10 Oct. 1867, 9.

^38^ All in *The Times* : ‘The Controversy Excited by Mr Skey’s Letter to the Editor’, 15 Oct. 1867, 6; F. C. Skey, ‘Athletics’, and G. Morrison, ‘Athletics’, both 21 Oct. 1867, 10; F. Willan, ‘Athletics’, and A Sexagenarian, ‘Athletics’, both 23 Oct. 1867, 10; Moderation, ‘Athletics’, 24 Oct. 1867, 9; P. P. Pennant, ‘Athletics’, 29 Oct. 1867, 9; Cornelia, ‘Athletics’, 30 Oct. 1867, 11.

^39^ Morus, ‘Measure of Man’; Mary Poovey, ‘Figures of Arithmetic, Figures of Speech: The Discourse of Statistics in the 1830s’, *Critical Inquiry* , xix (1993).

^40^ Allen Guttmann, *From Ritual to Record: The Nature of Modern Sports* , new edn (Columbia, 2004).

^41^ See, for example, T. C. Allbutt, ‘On Overwork and Strain of the Heart and the Aorta’, *Transactions of the Clinical Society of London* , vi (1873), 101.

^42^ George Kolb, *Physiology of Sport: Contributions towards the Physiology of a Maximum of Muscular Exertion, especially Modern Sports, as Rowing, Athletics, Gymnastics, Cycling, Swimming, etc.* , trans. George Kolb, 2nd edn (London, 1893); first pubd as George Kolb, *Beiträge zur Physiologie mazimalen Muskelarbeit, besonders des modernen Sports* (Berlin, 1888).

^43^ Kolb, *Physiology of Sport* , 49. The terms ‘professional’ and ‘amateur’ are hugely weighted. Much of the debate about the worth of sport can be best understood by seeing how these terms map onto ideas of class, education, expertise and other social markers. Such a discussion is outwith the remit of this article, but see Martin Polley, ‘The Amateur Ideal and British Sports Diplomacy, 1900–1945’, *Sport in History* , xxvi (2006); Marcus Collins, ‘The Fall of the English Gentleman: The National Character in Decline, *c.* 1918–1970’, *Historical Research* , lxxv (2002).

^44^ Kolb, *Physiology of Sport* , 31.

^45^ Roberta J. Park, ‘ “Cells or Soaring?” Historical Reflections on “Visions” of Body, Athletics, and Modern Olympism’, *International Journal of the History of Sport* , xxiv (2007); review of Kolb, *Physiology of Sport* , *British Medical Journal* , 25 Nov. 1893.

^46^ Review of Kolb, *Physiology of Sport* .

^47^ Fernand Lagrange, *Physiologie des exercices du corps* (Paris, 1889); trans. Fernand Lagrange as *Physiology of Bodily Exercise* (New York, 1898).

^48^ Charlotte Macdonald, *Strong, Beautiful and Modern: National Fitness in Britain, New Zealand, Australia and Canada, 1935–1960* (Vancouver, 2013); Ina Zweiniger-Bargielowska, *Managing the Body: Beauty, Health, and Fitness in Britain, 1880–1939* (Oxford, 2010); Michael Anton Budd, *The Sculpture Machine: Physical Culture and Body Politics in the Age of Empire* (Basingstoke, 1997).

^49^ Anaïs Bohuon and Antoine Luciani, ‘Biomedical Discourse on Women’s Physical Education in Sport in France, 1880–1922’, *International Journal of the History of Sport* , xxvi (2009), 580. See also R. A. Nye, ‘Degeneration, Neurasthenia, and the Culture of Sport in *Belle Époque* France’, *Journal of Contemporary History* , xvii (1982).

^50^ John Hoberman, *Mortal Engines: The Science of Performance and the Dehumanization of Sport* (London, 1992).

^51^ *Ibid.* ; but see also Paul Dimeo, *A History of Drug Use in Sport, 1876–1976: Beyond Good and Evil* (Abingdon, 2007), 18–19. See how Tissié is used in contemporary debates about doping in, for example, Verner Møller, *The Ethics of Doping and Anti-Doping: Redeeming the Soul of Sport?* (London, 2010), 97, 149.

^52^ Lagrange, *Physiology of Bodily Exercise* , 159.

^53^ *Ibid.* , 107–8, 183.

^54^ *Ibid.* , 159; Marland, *Health and Girlhood in Britain* ; Heggie, ‘Century of Cardiomythology’. See also Rob Beamish and Ian Ritchie, ‘From Fixed Capacities to Performance-Enhancement: The Paradigm Shift in the Science of “Training” and the Use of Performance-Enhancing Substances’, *Sport in History* , xxv (2005), although this article pushes the ‘shift’ Beamish and Ritchie identify back from the mid twentieth to the late nineteenth century.

^55^ Lagrange, *Physiology of Bodily Exercise* , 3.

^56^ Vanessa Heggie, *A History of British Sports Medicine* (Manchester, 2011); Vanessa Heggie, ‘Sport (and Exercise) Medicine in Britain: Healthy Citizens and Abnormal Athletes’, *Canadian Bulletin of Medical History* , xxviii (2011).

^57^ Justus Liebig, *Animal Chemistry: or, Organic Chemistry in its Application to Physiology and Pathology* , trans. William Gregory (London, 1842). For convenience I am going to use the word ‘protein’ throughout, although the original nineteenth-century works also use ‘nitrogenous foods’, ‘nitrogen-containing foods’, ‘proteid’ and other variations. The word ‘protein’, meaning ‘first stuff’, was coined by a Dutch chemist, Gerrit Jan Mulder (1802–80), in 1838, but not used consistently until the twentieth century: E. Brouwer, ‘Gerrit Jan Mulder, 1802–1880’, *Journal of Nutrition* , xlvi (1952).

^58^ Kenneth J. Carpenter, *Protein and Energy: A Study of Changing Ideas in Nutrition* (Cambridge, 1994); Graham Lusk, *The Elements of the Science of Nutrition* (Philadelphia, 1906), esp. ch. 5.

^59^ Carl von Voit, *Untersuchungen über den Einfluss des Kochsalzes, des Kaffees und der Muskelbewegungen auf den Stoffwechsel* (Munich, 1860); Carpenter, *Protein and Energy* .

^60^ David I. Trout, ‘Max Josef von Pettenkofer, 1818–1901: A Biographical Sketch’, *Journal of Nutrition* , cvii (1977).

^61^ M. von Pettenkofer and C. Voit, ‘Untersuchungen über den Stoffverbrauch des normalen Menschen’, *Zeitschrift für Biologie* , ii (1866).

^62^ Mikulas Teich, ‘Science and Food during the Great War: Britain and Germany’, in Harmke Kamminga and Andrew Cunningham (eds.), *Science and Culture of Nutrition, 1840–1940* (Amsterdam, 1995).

^63^ Aubin, ‘Hotel that Became an Observatory’.

^64^ A. E. Fick and J. Wislicenus, ‘On the Origin of Muscular Power’, *Philosophical Magazine and Journal of Science, London* , 4th ser., xxxi (1866).

^65^ Austin Flint Jr, ‘The Source of Muscular Power, as Deduced from Observations upon the Human Subject under Conditions of Rest and of Exercise’, *Journal of Anatomy and Physiology* , xii, 1 (1877).

^66^ Terrie M. Romano, ‘Gentlemanly versus Scientific Ideals: John Burdon Sanderson, Medical Education, and the Failure of the Oxford School of Physiology’, *Bulletin of the History of Medicine* , lxxi, 2 (1997); Soraya de Chadarevian, ‘Graphical Method and Discipline: Self-Recording Instruments in Nineteenth-Century Physiology’, *Studies in History and Philosophy of Science Part A* , xxiv (1993); E. M. Tansey, ‘ “. . . the Science Least Adequately Studied in England”: Physiology and the George Henry Lewes Studentship, 1879–1939’, *Journal of the History of Medicine and Allied Sciences* , xlvii (1992); Merriley Borell, ‘Instrumentation and the Rise of Modern Physiology’, *Science and Technology Studies* , v (1987); G. L. Geison, ‘Social and Institutional Factors in the Stagnancy of English Physiology, 1840–1870’, *Bulletin of the History of Medicine* , xlvi, 1 (1972).

^67^ Pavy, ‘Effect of Prolonged Muscular Exercise on the System, [Part I]’.

^68^ *Ibid.* , 319.

^69^ *Ibid.* , *passim* . See also F. W. Pavy, ‘The Effect of Prolonged Muscular Exercise on the System, [Parts II–V]’, *Lancet* , cvii, 2740–3 (1876); F. W. Pavy, ‘The Effect of Prolonged Muscular Exercise upon the Urine in Relation to the Source of Muscular Power, [Parts I–V]’, *Lancet* , cviii, 2778, 2780–2 (1876), cix, 2785 (1877).

^70^ Pavy, ‘Effect of Prolonged Muscular Exercise upon the Urine in Relation to the Source of Muscular Power, [Part I]’, 741.

^71^ All in *British Medical Journal* : J. Ashburton Thompson, ‘Mr Weston’s Pedestrian Feats’, 26 Feb. 1876; J. Ashburton Thompson, ‘Physiological Memoranda on E. P. Weston’s Third Walk’, 4 Mar. 1876; J. Ashburton Thompson, ‘Weston’s Fourth Walk’, 11 Mar. 1876.

^72^ Ashburton Thompson, ‘Weston’s Fourth Walk’, 335.

^73^ Pavy, ‘Effect of Prolonged Muscular Exercise on the System, [Part IV]’, 429.

^74^ Chris Renwick, ‘From Political Economy to Sociology: Francis Galton and the Social-Scientific Origins of Eugenics’, *British Journal for the History of Science* , xliv (2011).

^75^ James Hanley, ‘Edwin Chadwick and the Poverty of Statistics’, *Medical History* , xliv (2002); Christopher Hamlin, ‘Could You Starve to Death in England in 1839? The Chadwick–Farr Controversy and the Loss of the “Social” in Public Health’, *American Journal of Public Health* , lxxxv (1995). For other countries, see, for example, A. R. Ruis, ‘ “Children with Half-Starved Bodies” and the Assessment of Malnutrition in the United States, 1890–1950’, *Bulletin of the History of Medicine* , lxxxvii, 3 (2013).

^76^ For more on the socio-political construction of hunger, see James Vernon, *Hunger: A Modern History* (Cambridge, Mass., 2007). Although he locates the significant conceptual divide between malnutrition and undernutrition to 1920, this has been pushed back several decades by Heggie, ‘Lies, Damn Lies, and Manchester’s Recruiting Statistics’, into the nineteenth century.

^77^ Edward Smith and W. R. Milner, *Report on the Action of Prison Diet and Discipline on the Bodily Functions of Prisoners* (London, 1862); Edward Smith, ‘The Influence of the Labour of the Tread-Wheel over Respiration and Pulsation, and its Relation to the Waste of the System, and the Dietary of the Prisoners’, *Medical Times and Gazette* , new ser., xiv (1857); K. J. Carpenter, ‘Nutritional Studies in Victorian Prisons’, *Journal of Nutrition* , cxxxvi (2006).

^78^ See Edward Smith, ‘Report on the Food of the Poorer Labouring Classes in England’, in *Sixth Report of the Medical Officer of the Privy Council* (London, 1863); Edward Smith, *Dietaries for the Inmates of Work Houses: Report to the President of the Poor Law Board, and Poor Law Inspector* , Parliamentary Papers (Reports from Commissioners), 1866 (3660), xxxv.

^79^ Criticizing Liebig was an ongoing project for Smith: Edward Smith, ‘On Liebig’s Extract of Meat’, *Times* , 16 Oct. 1872, 6.

^80^ E. A. Parkes, ‘Further Experiments on the Effect of Diet and Exercise on the Elimination of Nitrogen’, *Proceedings of the Royal Society of London* , xix (1871).

^81^ Edmund A. Parkes, *A Manual of Practical Hygiene: Prepared Especially for Use in the Medical Service of the Army* (London, 1864), esp. 145 n.

^82^ H. W. Bywaters, ‘F. W. Pavy’, *Biochemical Journal* , x (1916).

^83^ W. O. Atwater and C. F. Langworthy, *A Digest of Metabolism Experiments in which the Balance of Income and Outgo Was Determined: US Department of Agriculture Bulletin No. 45* (Washington, 1897).

^84^ Robin Wolfe Scheffler, ‘The Power of Exercise and the Exercise of Power: The Harvard Fatigue Laboratory, Distance Running, and the Disappearance of Work, 1919–1947’, *Journal of the History of Biology* , xlviii (2015); Robin Wolfe Scheffler, ‘The Fate of a Progressive Science: The Harvard Fatigue Laboratory, Athletes, the Science of Work and the Politics of Reform’, *Endeavour* , xxxv (2011).

^85^ On the latter, see D. Riley, ‘Physical Deterioration of School Children in the Late Nineteenth and Twentieth Centuries and Suggested Remedies’ (Univ. of Manchester M.Ed. thesis, 1973); Welshman, ‘Physical Culture and Sport in Schools in England and Wales, 1900–40’.

^86^ See n. 66 above; Carin Berkowitz, ‘Disputed Discovery: Vivisection and Experiment in the Nineteenth Century’, *Endeavour* , xxx (2006); Richard D. French, *Antivivisection and Medical Science in Victorian Society* (Princeton, 1975).

^87^ Robert Tattersall, ‘Frederick Pavy (1829–1911) and his Opposition to the Glycogenic Theory of Claude Bernard’, *Annals of Science* , liv (1997).

^88^ Pavy, ‘Effect of Prolonged Muscular Exercise on the System, [Part I]’, 320. For more on Weston’s participation, see Harris, Harris and Marshall, *Man in a Hurry* , which takes this material very seriously, unlike many sports histories.

^89^ On the gendering of the normal male body, see Londa Schiebinger, ‘Why Mammals Are Called Mammals: Gender Politics in Eighteenth-Century Natural History’, *American Historical Review, xcviii, 2* (1993).

